# Postmarketing safety surveillance data reveals antidepressant effects of botulinum toxin across various indications and injection sites

**DOI:** 10.1038/s41598-020-69773-7

**Published:** 2020-07-30

**Authors:** Tigran Makunts, Marc Axel Wollmer, Ruben Abagyan

**Affiliations:** 1 Skaggs School of Pharmacy and Pharmaceutical Sciences, University of California San Diego, La Jolla, CA USA; 2 Oak Ridge Institute of Science and Education Fellowship at Office of Clinical Pharmacology, United States Food and Drug Administration, Silver Spring, MD USA; 3 Asklepios Clinic North-Ochsenzoll, Asklepios Campus Hamburg, Medical Faculty, Semmelweis University, Hamburg, Germany

**Keywords:** Depression, Therapeutics, Adverse effects, Depression, Neurology, Neurological disorders, Psychiatric disorders, Bioinformatics

## Abstract

The World Health Organization estimates the number of people suffering from depression to be over 264 million. Current monoamine transmission modulating therapeutics, even with proper adherence and acceptable tolerability, are not effective for nearly one third of the patients, leading clinicians to explore other therapeutic options such as electroconvulsive therapy, transcranial magnetic stimulation, ketamine infusions, and, more recently, glabellar botulinum toxin, BoNT, injections. The scale and mechanism of antidepressant action of BoNT is unclear and maybe hypothetically attributed to the disruption of proprioceptive facial feedback reinforcing negative emotions. Here we verify the antidepressant effect of botulinum toxin by analysis of over 40 thousand BoNT treatment reports out of thirteen million postmarketing safety reports in the FDA Adverse Event Reporting System, FAERS. The results of the analysis indicate that patients who received BoNT injections to treat hyperhidrosis, facial wrinkles, migraine prophylaxis, spasticity, and spasms, had a significantly lower number of depression reports when compared to patients undergoing different treatments for the same conditions. These findings suggest that the antidepressant effect of BoNT is significant, and, surprisingly, is observed for a broad range of injection sites.

## Introduction

Depression is one of the top three contributors to the global disease burden with an estimated lifetime prevalence rate of 8–12% in industrialized countries^1,2^. In a recent Global Health Metrics study, depressive disorders were one of the top three contributors to the years-lost-to-disability (YLD) measure^3^.

Besides psychotherapy, the standard approach to treating depression mainly consists of serotonin, dopamine, or norepinephrine pathway modulating therapeutics at present. However, side effects, delayed onset of the beneficial action, fear of drug-dependence, and limited efficacy, frequently lead to poor adherence and discontinuation of the treatment^4–6^. Even with acceptable tolerability and adherence, nearly one third of the patients don’t respond to any antidepressants, including selective serotonin reuptake inhibitors (SSRI), dopamine-norepinephrine reuptake inhibitors (DNRI), and/or serotonin-norepinephrine reuptake inhibitors (SNRI)^7^, prompting clinicians to consider other therapeutic options for primary or adjunct treatments such as electroconvulsive therapy^8^, transcranial magnetic stimulation^9^, ketamine administation^10^, and facial injections of botulinum toxin^11,12^.

In earlier studies we performed an Inverse-Frequency Analysis of postmarketing pharmacovigilance cases reported to the United States Food and Drug Administration through MedWatch^13^ and noticed significantly lower depression rates for ketamine, minocycline, NSAIDs, and botulinum toxin (BoNT)^14,15^. Rates of depression in the BoNT cohort were of particular interest, since at the time there were several clinical trials and case studies indicating its possible efficacy in treating depression.

BoNT was first approved for therapeutic use in 1989 for eye muscle disorders, such as strabismus and blepharospasm^16–18^. It was later approved for a number of indications including cosmetic use^19^, hyperhidrosis^20^, migraine prophylaxis^21^, neurogenic bladder disorder^22^, overactive bladder^23^, urinary incontinence^23^, and spasticity^24^. BoNT is used off-label for achalasia^25^, and sialorrhea^26^. It is considered generally safe with most common side effects being hypersensitivity, injection side reactions, and other side effects specific to the injection site and indication^16^. BoNT’s mechanism of action is primarily attributed to muscle paralysis by blocking presynaptic acetylcholine release into neuromuscular junctions^27^.

In the last decade, several randomized double-blind placebo-controlled clinical trials^11,28–30^, case series^31^, and an open-label study^32^ have demonstrated significant efficacy of BoNT injections into the glabellar region of the face in treating depression. These studies were followed by a larger, industry-sponsored Phase II trial with 258 participants however the results were mixed^12^. Although there was a decrease in depression rating scores and numeric superiority of BoNT over placebo, the results did not meet the primary endpoint criteria.

Collectively, the evidence from these studies implied some variability in efficacy of BoNT use in depression. The interpretation of the studies is limited by several factors: (1) although several studies have been conducted, the overall number of patients is still small; and (2) because of the obvious cosmetic effects associated with BoNT treatment, it was impossible to reliably blind patients for allocation to the BoNT or placebo treatment arm, which may have inflated the impact of expectations (placebo effects) and disappointment (nocebo effects) over the clinical effect. Moreover, it is unclear to what extent the cosmetic effect may have contributed to the observed improvement in the symptoms of depression. Furthermore, (3) the mechanism by which BoNT exerts its antidepressant effect remains unknown. The rationale of many studies was the facial feedback hypothesis. On this theoretical basis BoNT injections were exclusively placed in the glabellar region to paralyze key muscles (*corrugator* and *procerus* muscles; “grief muscles”) for the expression of negative emotions (sadness, fear, anger) and thereby disrupt proprioceptive feedback from the face to the brain that maintains and reinforces theses emotions. However, it is possible that the observed antidepressant effects were attained via entirely different mechanisms of action^33–36^.

To address these limitations we analyzed over forty five thousand clinical reports of adverse events (AEs) resulting from BoNT treatments not only for cosmetic, but also for various other indications and respective injection sites including migraine, upper and lower limb spasms and spasticity, neck muscle disorders, blepharospasm, sialorrhea, and bladder injections for urinary and neurological disorders. With this approach we (1) accessed a larger number of reports, (2) eliminated expectation effects regarding antidepressant action, and (3) introduced a specificity control for the glabellar injection site.

Here we expanded the evidence on the antidepressant effect of BoNT and revealed that it may be independent from the site of administration.

## Methods

### FDA adverse event reporting system (FAERS)

AERS/FAERS supports post-marketing surveillance and AEs for drugs and biologics submitted to the United States Food and Drug Administration (FDA) through MedWatch^13^. The reporting is mostly voluntary and is done by physicians, legal representatives, nurses, pharmacists, other healthcare professionals, and patients. In case where the AE is reported to the manufacturer, the latter is legally obligated to forward the AE report to the AERS/FAERS system.

The study used over thirteen million reports available from the FDA AERS and FAERS data sets. At the time of the analysis the FAERS set contained data from September 2012 to December 2019, and its older version, AERS, set contained data from January 2004 to August 2012. The reports were used to run a retrospective Inverse-Frequency Analysis on the drugs of interest. Both AERS and FAERS data sets are available online at: https://www.fda.gov/Drugs/GuidanceComplianceRegulatoryInformation/Surveillance/AdverseDrugEffects/ucm082193.htm.

### Combining and normalizing AERS/FAERS data

Each quarterly AERS and FAERS data set was individually downloaded and saved in dollar-separated text (.txt) format. The format of the data has been changed several times and is not uniform in all quarters/years. The data was modified and standardized to create a consistent table structure where missing columns were added with missing values. The data is submitted to AERS/FAERS through MedWatch^13^ from around the world with their respective country specific medication brand names.

### Study outcomes

20,317 uniquely worded outcomes were observed in AERS/FAERS. The outcome of interest was defined as an adverse event of a depression or depressive disorder related adverse event, defined in AERS/FAERS by the following MedDRA^37^ terms: depression, treatment resistant depression, depressed mood, major depression, adjustment disorder with depressed mood, depressive symptom, adjustment disorder with mixed anxiety and depression, agitated depression, persistent depressive disorder, depression suicidal, adjustment disorder with anxiety and depressed mood, suicidal ideation, suicide attempt, suicidal behavior.

### Cohort selection

**Figure 1:**
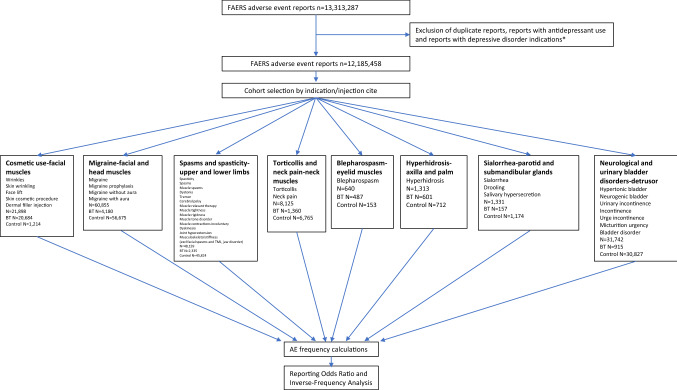
Analysis flow chart, and inclusion/exclusion terms for cohort selection, used in adverse event rate comparison between botulinum toxin and control cohorts.

**Figure 2:**
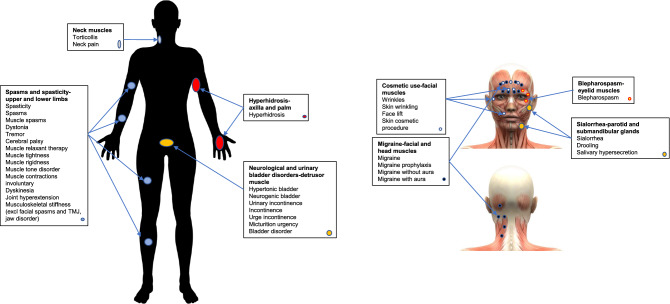
Study cohorts by indication and injection site. Christos Georghiou/shutterstock.com, decade3d—anatomy online/shutterstock.com.

A total of 13,313,287 reports until year 2020 were collected. Reports related to patients taking any antidepressants or reports containing depression as one of the indications were eliminated to avoid confounding effects (see details in S1 and S2 Appendices) resulting in 12,185,458 reports. Reports with botulinum toxin (defined as OnabotulinumtoxinA, AbobotulinumtoxinA, IncobotulinumtoxinA, and RimabotulinumtoxinB)^38^ were analyzed to define eight cohorts corresponding to the most frequent indications or injection sites (Figs. 1, 2). The resulting cohorts were the following: (1) *Cosmetic use—facial muscles* (wrinkles, skin wrinkling, face lift, skin cosmetic procedure, dermal filler injection); (2) *Migraine—facial and head muscles* (migraine, migraine prophylaxis, migraine without aura, migraine with aura); (3) *Spasms and Spasticity—upper and lower limbs* (spasticity, muscle spasms, dystonia, tremor, cerebral palsy, muscle relaxant therapy, muscle tightness, muscle rigidness, muscle tone disorder, muscle contractions involuntary, dyskinesia, joint hyperextension, musculoskeletal stiffness), disorders related to facial muscles such as facial spasms, temporomandibular joint disorder and jaw disorder were excluded; (4) *Torticollis and neck pain—neck muscles*; (5) *Blepharospasm—eyelid muscles*; (6) *Hyperhidrosis—axilla and palm*; (7) *Sialorrhea—parotid and submandibular glands* (drooling, salivary hypersecretion); (8) *Neurological and urinary bladder disorders—detrusor muscle* (hypertonic bladder, neurogenic bladder, urinary incontinence, incontinence, urge incontinence, micturition urgency, bladder disorder (Figs. 1, 2). Each cohort was separated into two sub-cohorts: BoNT (exposed) and non-BoNT (control) (S3 Appendix). Frequencies of depression and related AEs were calculated for patients in each sub-cohort. Reporting odds ratios (ROR) were calculated to identify any protective effect through Inverse-Frequency Analysis.

### Statistical analysis

The statistical analysis of the FAERS and other safety surveillance data is well established, it includes frequencies, reporting odds ratios and 95% confidence intervals, and is fully described in many publications^39–41^. Here is a summary of the formulae.

1. Descriptive statistics (3a): Frequency for each side effect was calculated by the equation:
  $$ {\text{Reporting frequency}} = {\text{No}}.{\text{ of records with depression AEs }}/{\text{ No}}.{\text{ of patient records}} $$
2. Comparative statistics (3b): Depression related report rates were compared via the Reporting Odds Ratio (ROR) using the following equations:
  $$ {\text{ROR }} = \, \left( {{\text{a }}/{\text{ b}}} \right) \, / \, \left( {{\text{c }}/{\text{ d}}} \right) $$

a = No. in exposed group with AE, b = No. in exposed group with no AE, c = No. in control group with the AE, d = No. in control group with no AE.

Standard Error (SE) of the LnROR value was calculated by the following equation:

$$ {\text{SELnROR }} = \, \sqrt {\left( {{1}/{\text{a }} + { 1}/{\text{b }} + { 1}/{\text{c }} + { 1}/{\text{d}}} \right)} $$

Error bars were computed using 95% confidence intervals.

$$ {95}\% {\text{ CI }} = \exp \left( {{\text{LnROR }} - { 1}.{96 } \times {\text{ SELnROR }}} \right) {\text{to}}\,\exp \left( {{\text{LnROR }} + { 1}.{96 } \times {\text{ SELnROR }}} \right) $$

Haldane-Anscombe correction was used in small sample cohorts with zero reports of interest^42^.

## Results

### Botulinum toxin: depression and depressive disorder related AEs

**Figure 3:**
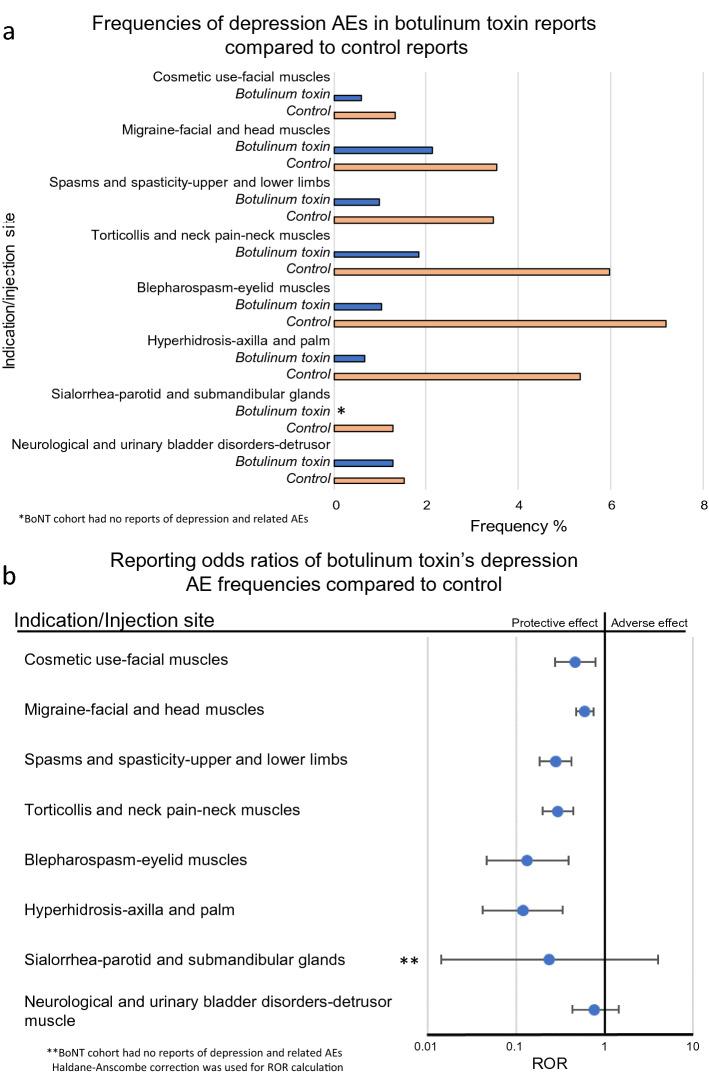
Frequencies and reporting odds ratios (RORs) of depression events. (**a**) Frequencies of depression events for patients administered botulinum toxin for cosmetic use (BoNT N = 20,684, control N = 1,214), migraine (BoNT N = 4,180, control N = 56,675), spasms and spasticity, excluding facial muscles (BoNT N = 2,335, control N = 45,824), torticollis (BoNT N = 1,360, control N = 6,765), blepharospasm (BoNT N = 487, control N = 153), hyperhidrosis (BoNT N = 601, control N = 712), sialorrhea (BoNT N = 157, control N = 1,174), neurological and urinary bladder disorders (BoNT N = 915, control N = 30,827). (**b**) Reporting odds ratios were calculated comparing frequencies of depression reports in patients administered botulinum toxin for each indication and respective control sub-cohorts. Ranges represent 95% confidence intervals (95% CI) (see “Methods”). *BoNT* botulinum toxin, *AE* adverse event, *ROR *reporting odds ratios.

Patients who were administered BoNT had a significantly lower incidence of depression and depression-related AE reports, compared to the control groups. It was observed not only for *cosmetic use in facial muscles* (reporting odds ratios (ROR) 0.46, 95% confidence interval (CI) [0.27, 0.78]), but also for most other indications and injection sites including:, *migraine—facial and head muscles* (0.60 [0.48, 0.74]), *spasms and spasticity—upper and lower limbs*, excluding facial muscles (0.28 [0.18, 0.42]), *torticollis and neck pain—neck muscles* (0.30 [0.20, 0.44]), *blepharospasm—eyelid muscles* (0.13 [0.05, 0.39]), and *hyperhidrosis—axilla and palm* (0.12 [0.04, 0.33]). In a more dramatic manifestation of the antidepressant effects, there were no reports of depression or related AEs in the BoNT *sialorrhea—parotid and submandibular glands* sub-cohort. However, due the small size of sub-cohorts for this indication, the reduced ROR value derived from 0/157 and 15/1,159 was evaluated as not-significant at 95% CI level after the Haldane-Anscombe correction^42^ was applied (0.24 [0.01, 3.99]). Interestingly there was also a decrease in depression reports where BoNT was injected into the detrusor muscle in the *neurological and urinary bladder disorders* cohort, but the reduced ROR value did not reach statistical significance (0.77 [0.43, 1.43]) (Fig. 3).

## Discussion

### Antidepressant effect of BoNT does not depend on the injection site

In our study we analyzed 174,243 reports divided into eight BoNT-treatment-related cohorts to evaluate the significance and the dependence of antidepressant effect on the site of administration. We confirmed the antidepressant effect of facial BoNT injections as they were investigated in previous clinical trials. These trials were flawed by the impossibility to reliably blind patients for treatment allocation and control for placebo and nocebo effects. Our study overcomes these objections as it shows antidepressant effects of BoNT injections in the absence of expectations in this regard.

To our surprise the observed antidepressant effect RORs were significantly reduced for six out of eight indications/injection sites. In the remaining two cohorts, the observed values were reduced too, but the 95% CI ranges did not reach the threshold for statistical significance. Although the facial feedback/emotional proprioception hypothesis^43^ is a plausible and substantiated mechanism by which botulinum toxin may exert its antidepressant effects, our findings suggest that it may act through a more complex mechanism.

### Plausible mechanisms of BoNT antidepressant effect

While the effect established in our analysis cannot be fully explained at the molecular level at this point, there are several possibilities for a unifying mechanism of the antidepressant effect of BoNT in the different indications:

#### Transneuronal transport

BoNT may undergo a targeted, transneuronal transport to CNS structures that are involved in the regulation of mood and emotions. There is experimental evidence of axonal and trans-synaptic transport of peripherally injected BoNT into the CNS, if injected in high doses^44,45^. There is also clinical evidence that CNS BoNT effects may be medically relevant in humans^46^. Studies on peripheral injections of BoNT for spasms and spasticity have reported that the toxin may go beyond the muscle groups of the injection site and affect the *opposing* muscle groups and reflexes through more complex neurocircuitry changes^47^. Thus, transneuronal transport to remote sites in the CNS is in principle conceivable. However, it is unlikely that BoNT from all the different injection sites may be targeted to the same structures in the CNS to induce comparable antidepressant effects. Alternatively, BoNT injections at different injections sites may induce similar neuroplastic processes without long-distance transneuronal transport that may account for the antidepressant effect^48^.

#### Systemic distribution

In theory, BoNT may also accomplish its antidepressant effect after systemic distribution. However, the amount of BoNT in the circulation that is available for a systemic effect is probably very low, especially after injection of small muscles with low doses of BoNT^49^. In this scenario, a dose-dependent antidepressant effect would be expectable. However, the reported rates of depression after BoNT treatment of e.g. blepharospasm (low dose) and limb spasticity (high dose) are similar and the antidepressant effect compared to the control is even stronger in the former condition.

#### Distributed muscle stress memory

The reciprocal, mutually reinforcing interrelation of muscle action and mood is not confined to facial feedback mechanisms. Muscle tension in various body regions is a frequent symptom in depression and may be both a psychomotor manifestation and a booster of depressed mood^50,51^. Hence, progressive muscle relaxation (PMR) is a well-established relaxation method in psychiatric treatment that relies on the induction of mental relaxation via tension and subsequent relaxation of skeletal muscles. The investigated conditions of increased muscle tone, especially torticollis and blepharospasm are associated with depression, and it may be speculated that proprioceptive feedback from the affected muscles may be causally involved in this association^52–54^. Conversely, reduction in muscle tone as result of BoNT injection may counteract depression in analogy to the mood-lifting effect of glabellar BoNT injections. Of note, the BoNT injection scheme for chronic migraine and frequently also for blepharospasm comprises the corrugator muscles. Interestingly, in the latter indication, a stronger reduction in depressive symptoms has been observed after BoNT treatment involving the glabellar muscles. The feedback concept may be extended to a vegetative feedback mechanism: Hyperhidrosis is strongly associated with depression and it is conceivable that increased sweating is not only a vegetative manifestation of stress, but may also have a stress- and possible depression-enhancing feedback effect^55^.

#### Efficacy in the primary indication treatment

In the investigated conditions, BoNT may be superior to the treatment options that were taken as comparators, both in terms of efficacy and tolerability. As these conditions are chronic and burdensome, they are associated with secondary psychiatric comorbidities like depression, which are prevented by the relief in the primarily treated condition (e.g. for blepharospasm there is no really coequal oral medication^56^). A specific antidepressant effect of BoNT may be overestimated in these conditions because of differential relief from the burden of disease between the BoNT and the control group. We provide an extensive list for the treatments for each of the indication cohorts in the Supplementary Information S3. Regrettably, we were not able to quantify the efficacy of the treatments from the analyzed safety data sets due to the absence of such information in the safety reports.

In the cosmetic indication there is no burden of disease. Accordingly, the comparator group shows lower depression rates than the comparator groups of other indications. Hence, the antidepressant effect of BoNT may be underestimated in this indication because of a bottom effect for the occurrence of depression and depression-related symptoms in this cohort.

Most of the evidence on the antidepressant effect of BoNT is from studies where the injection was administered to the glabellar region. Interestingly, there is evidence that when BoNT is administered for crow’s feet in *orbicularis oculi* muscles, the depression scores increase^57^, possibly due to prevention of the Duchenne smile. In the analyzed data set, the specific injection muscles were not specified, and the broad indication for wrinkles, cosmetic use etc. was listed instead.

The treatment in the comparator groups may have depression as a side effect, which may inflate the antidepressant effect of BoNT. To eliminate any concern about the biased nature of the control cohorts we evaluated the concurrent treatments in the all cohorts. The reports with explicit relation to depression treatments were removed, while the remaining treatments were balanced between the BoNT and control cohorts in their indirect depression related side effects (S1–S3 Appendix).

In summary, our findings confirm the antidepressant effect of facial BoNT injections that was described in a series of clinical studies. Based on 20,684 BoNT-treated cases and 1,214 control subjects from the FAERS database, we show that cosmetically motivated facial BoNT injections involving the glabellar region have a preventive effect against the occurrence of depression or depressive symptoms. This also implies a potential of BoNT as a therapeutic in the (relapse)-prevention of depression. These findings are further corroborated by a similar antidepressant effect we find for BoNT injections, also involving the glabellar region, in a large number of migraine patients, for whom the antidepressant effects of BoNT have been observed before, too. Conditionally, this also applies for the findings for blepharospasm. However, the situation is a bit more complex and ambiguous here. On the one hand, the lateral part of the *musculus orbicularis oculi* is involved in Duchenne’s smile and, thus, the expression of positive emotions. Accordingly, its paralysis in the cosmetic treatment of crow’s feet wrinkles by injection of BoNT has been associated with increased depression scores^57^. On the other hand, depression associated with blepharospasm is relieved if the affected muscles are injected with BoNT, and the antidepressant effect may be more pronounced if the treatment involves the glabellar region^58,59^.

Moreover, we show that BoNT injections in body regions other than the glabella also have a protective effect against depression in several burdensome medical conditions that are associated with a high risk for comorbid depression. This is also in line with the previous literature^60–62^. This implies the possibility that in addition to, or even instead of, the proposed facial feedback mechanism other mechanisms of action may account for the antidepressant effect of BoNT. The growing evidence of the non-neuromuscular junction effects of the toxin suggests that BoNT’s antidepressant effect may be attributable to a currently unknown global pharmacological effects of BoNT within the central nervous system. Future investigations into the mechanisms by which BoNT exerts its antidepressant effect should consider this possibility. In studies where BoNT was used to treat migraine, BoNT showed improved reduction of symptoms of depression and anxiety^63,64^, implying the association of pain with depression as an explanation for the BoNT’s efficacy in depression^65,66^, but it is noteworthy that the observed antidepressant effects were comparable in several indications not associated with pain (Fig. 3). Separating the pain vs. proprioception hypotheses is challenging since in migraine prophylaxis treatment, BoNT injection sites do include the glabellar region, which may introduce a facial feedback effect.

## Conclusion

Our findings show that the antidepressant effect of BoNT administered for various indications goes beyond the control of the intended disease states and does not depend on the location of the injection. In this respect BoNT proves superior to the alternative treatment options summarized in the respective comparator group.

Upcoming phase III clinical studies will decide if glabellar BoNT injections may be approved as a treatment for depression. Until then, our findings support the application of BoNT for this indication and, at the same time, point out the potential for further optimization of the treatment based on a better understanding of the mechanism of action.

### Study limitations

FAERS/AERS reporting is voluntary and open to the public. The investigated data sets represent only a subset of actual cases and the frequencies should not be confused with population incidences. Additionally, the reporting to FAERS/AERS may be biased by legal, scientific variables and newsworthiness^67^ and are often underreported^68^. To address these limitations, disproportionality analysis with reporting odds ratios and 95% CI was used to assess the significance of the difference between the sub-cohorts. Other limitations to consider include occasionally missing demographic variables, treatment doses and durations, and comprehensive medical records as well as bias associated with the comparator (differential efficacy, undetected differences between patients treated with the substance of interest and the comparator).

We excluded all the reports where the comorbidities included depression or depressive disorders and any reports where a known antidepressant was used, however, concurrent medications, including over-the-counter drugs and supplements, and present comorbidities may be underreported which may affect the analysis results. This limitation is present in all levels of clinical research including case studies and controlled trials. Since reports with depression as a preexisting comorbidity were excluded, the antidepressant effects described here are preventive, not therapeutic effects.

## Supplementary information

Supplementary file 1

## Data Availability

The data sets are de-identified and made available to the public online by the United States Food and Drug Administration. Institutional Review Board requirements do not apply under 45 CFR 46.102. https://www.fda.gov/drugs/questions-and-answers-fdas-adverse-event-reporting-system-faers/fda-adverse-event-reporting-system-faers-latest-quarterly-data-files.
