# Supplementary material for: Postmarketing safety surveillance data reveals antidepressant effects of botulinum toxin across various indications and injection sites
Source: Sci Rep. 2020 Jul 30;10:12851. doi: 10.1038/s41598-020-69773-7 (PMC7393507; doi:10.1038/s41598-020-69773-7)
Supplement: Supplementary file 1 [file 41598_2020_69773_MOESM1_ESM.docx]

**Postmarketing safety surveillance data reveals antidepressant effects of botulinum toxin across various indications and injection sites**

Tigran Makunts^1,2^, M. Axel Wollmer^3^, Ruben Abagyan^1*^

^1^ Skaggs School of Pharmacy and Pharmaceutical Sciences, University of California San Diego, La Jolla, California

^2^Oak Ridge Institute of Science and Education fellowship at Office of Clinical Pharmacology, United States Food and Drug Administration

^3^ Asklepios Clinic North - Ochsenzoll, Asklepios Campus Hamburg, Medical Faculty, Semmelweis University, Germany

Correspondence to RA: [rabagyan@health.ucsd.edu](mailto:rabagyan@health.ucsd.edu)

**Supplementary information**

**S1 Appendix. Excluded antidepressant list.**

 Sertraline, fluoxetine, citalopram, escitalopram, paroxetine, fluvoxamine, venlafaxine, desvenlafaxine, duloxetine, levomilnacipran , milnacipran, amitriptyline, amoxapine, clomipramine, desipramine, nortriptyline, doxepin, imipramine, protriptyline, trimipramine, maprotiline, bupropion, vilazodone, nefazodone,  trazodone, vortioxetine, mirtazapine, isocarboxazid, phenelzine, tranylcypromine, selegiline, milnacipran, tofenacin, etoperidone, amitriptylinoxide, dibenzepin, dimetacrine, dosulepin , adapin, lofepramine, melitracen, nitroxazepine, noxiptiline, pipofezine, butriptyline, demexiptiline, imipraminoxide, iprindole, metapramine, propizepine, quinupramine, mianserin, setiptiline, caroxazone, metralindole, moclobemide, pirindole, toloxatone, eprobemide, minaprine, ketamine.

**S2 Appendix**. **Excluded depressive disorder related indications/comorbidities**

Depression, treatment resistant depression, depressed mood, major depression, adjustment disorder with depressed mood, depressive symptom, adjustment disorder with mixed anxiety and depression, agitated depression, persistent depressive disorder, depression suicidal, adjustment disorder with anxiety and depressed mood, suicidal ideation, suicide attempt, suicidal behavior.

**S3 Appendix. List of concurrent medications in BoNT and control groups**

| **Indication** | **BoNT cohort concurrent medications (>1% reported)** | **Control cohort medications**  **(>1% reported)** |
| --- | --- | --- |
| Cosmetic use | Hyaluronic acid, synthetic calcium hydroxyapatite | Tretinoin, hyaluronic acid, adapalene, hydroquinone, clobetasol propionate, deoxycholic acid, epinephrine with lidocaine, fibrinogen (BAC2) and thrombin |
| Migraine | Sumatriptan, topiramate, gabapentin, rizatriptan, ibuprofen, propranolol, eletriptan, acetaminophen/butalbital/caffeine,  zolmitriptan, aspirin, tizanidine, diclofenac, ergocalciferol, valproic acid, erenumab-aooe, galcanezumab-gnlm, tramadol, ketorolac, pregabalin, naproxen | Sumatriptan, erenumab-aooe, topiramate, ibuprofen, eletriptan, acetaminophen, gabapentin, rizatriptan, propranolol, acetaminophen/caffeine, acetaminophen/butalbital/caffeine,  zolmitriptan, aspirin, tizanidine, diclofenac, ergocalciferol, valproic acid, galcanezumab-gnlm, tramadol, ketorolac, pregabalin, naproxen, acetaminophen/hydrocodone, naratriptan, naproxen/sumatriptan, fentanyl |
| Spasms and spasticity | Baclofen, gabapentin, diazepam, aspirin, tizanidine, clonazepam, valproic acid, pregabalin, levetiracetam, cyclobenzaprine, lamotrigine, topiramate, dalfampridine, carbamazepine, zolpidem, midazolam, carbidopa/levodopa | Baclofen, gabapentin, cyclobenzaprine, pregabalin, diazepam, tizanidine, clonazepam, lorazepam, zolpidem, tetrabenazine, primidone, valproic acid, dalfampridine, carisoprodol, topiramate, carbidopa/levodopa |
| Torticollis and neck pain | Clonazepam, gabapentin, diazepam, baclofen, tizanidine, lorazepam, cyclobenzaprine, phenobarbital, trihexyphenidyl, valproic acid, pregabalin | Pregabalin, gabapentin, cyclobenzaprine, diazepam, tizanidine, clonazepam, alprazolam, carisoprodol, baclofen, lorazepam, topiramate |
| Blepharospasm | Clonazepam, lorazepam, diazepam, trihexyphenidyl | Clonazepam, carbamazepine, tetrabenazine, pregabalin, lorazepam, cyclobenzaprine, baclofen, trihexyphenidyl, alprazolam, gabapentin |
| Hyperhidrosis | none | Glycopyrronium, aluminum chloride hexahydrate with anhydrous ethyl alcohol |
| Sialorrhea | Glycopyrronium, scopolamine, hyoscyamine | Scopolamine, pirenzepine, glycopyrronium, amisulpride, trihexyphenidyl |
| Neurological and urinary bladder disorders | Oxybutynin, solifenacin, mirabegron, tolterodine, trospium, tamsulosin, fesoterodine | Solifenacin, oxybutynin, mirabegron, tolterodine, fesoterodine, tamsulosin, darifenacin, trospium, doxazosin |
